# Carriers of a novel frame-shift insertion in *WNT16a* possess elevated pancreatic expression of TCF7L2

**DOI:** 10.1186/1471-2156-14-28

**Published:** 2013-04-23

**Authors:** Eric W Howard, Latonya F Been, Megan Lerner, Daniel Brackett, Stan Lightfoot, Elizabeth C Bullen, Dharambir K Sanghera

**Affiliations:** 1 Department of Cell Biology, College of Medicine, University of Oklahoma Health Sciences Center, Oklahoma City, OK, USA; 2 Department of Pediatrics, College of Medicine, University of Oklahoma Health Sciences Center, 940 Stanton L. Young Blvd., Rm 317 BMSB, Oklahoma City, OK, 73104, USA; 3 Department of Surgery, College of Medicine, University of Oklahoma Health Sciences Center, Oklahoma City, OK, USA; 4 Veteran Affairs, VA Medical Center, Oklahoma City, OK, USA

**Keywords:** β-cat /TCF7L2 signaling, Wnt16a, Exome sequencing, Insertion polymorphism, *TCF7L2* gene variants, Gene expression, Pancreatic β-cells, Type 2 diabetes

## Abstract

**Background:**

The discovery of *TCF7L2* as a global type 2 diabetes (T2D) gene has sparked investigations to explore the clinical utility of its variants for guiding the development of new diagnostic and therapeutic strategies. However, interpreting the resulting associations into function still remains unclear. Canonical Wnt signaling regulates β-catenin and its binding with TCF7L2, which in turn is critical for the production of glucagon-like peptide-1 (GLP-1). This study examines the role of a novel frame-shift insertion discovered in a conserved region of *WNT16a*, and it is proposed that this mutation affects T2D susceptibility in conjunction with gene variants in *TCF7L2*.

**Results:**

Our results predicted that the insertion would convert the upstream open reading frame in the Wnt16a mRNA to an alternative, in-frame translation initiation site, resulting in the prevention of nonsense-mediated decay, leading to a consequent stabilization of the mutated WNT16a message. To examine the role of Wnt16a in the Wnt signaling pathway, DNA and serum samples from 2,034 individuals (48% with T2D) from the Sikh Diabetes Study were used in this investigation. Prevalence of Wnt16a insertion did not differ among T2D cases (33%) and controls (32%). However, there was a 3.2 fold increase in Wnt16a mRNA levels in pancreatic tissues from the insertion carriers and a significant increase (70%, p < 0.0001) in luciferase activity in the constructs carrying the insertion. The expression of TCF7L2 mRNA in pancreas was also elevated (~23-fold) among the insertion carriers (p=0.003).

**Conclusions:**

Our results suggest synergistic effects of *WNT16a* insertion and the at-risk ‘T’ allele of TCF7L2 (rs7903146) for elevating the expression of *TCF7L2* in human pancreas which may affect the regulation of downstream target genes involved in the development of T2D through Wnt/β-catenin/TCF7L2 signaling pathway. However, further studies would be needed to mechanistically link the two definitively.

## Background

Transcription factor 7-like 2 (TCF7L2) has been strongly linked to type 2 diabetes (T2D) susceptibility, with an elevated genetic predisposition accounting for 20% of T2D cases [1]. The association of common intronic variants in the *TCF7L2* gene with the increased susceptibility for T2D has been extensively documented in major ethnic groups of the world by several different investigators [2]. Meta-analysis of the published studies estimated the odds ratio (OR) of 1.46 (p=5.4x10^-140^) [3]. *TCF7L2* polymorphisms were also significantly linked to diabetes risk in our own studies in Asian Indian Sikhs [4,5]. Indeed, our recent Sikh genome-wide association study (GWAS) and meta-analyses in Sikhs (n=7,329/3,354 cases) and South Asians (n=47,303/19, 482 cases) showed a robust association of *TCF7L2* (rs7903146), with OR 1.5 (p=7.8x10^-19^) and OR 1.13 (p=6.1x10^-25^) in Sikhs and South Asians, respectively [6]. However, despite extensive replication, no study has unequivocally demonstrated the underlying molecular mechanism of this association. Little is known about the clinical role of *TCF7L2* in T2D beyond progression from impaired glucose tolerance to diabetes [7].

**Figure 1:**
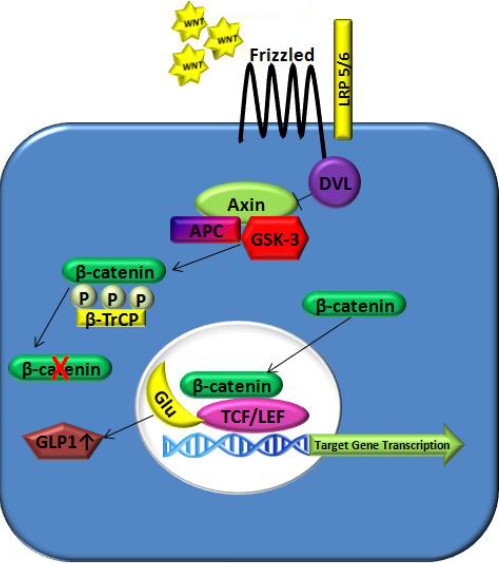
Wnt signaling pathway in diabetes mellitus.

Various *in vitro* and *in vivo* studies have shown that several components of the Wnt pathway are involved in β-cell proliferation [8], insulin secretion and cholesterol metabolism [9], and production of glucogon-like peptide-1 (GLP-1) [10]. Wnts are secreted glycoproteins with a well-established role in the early stages of development through adulthood [11]. Wnts bind to frizzled and LRP receptors, which, in turn, inactivate the degradation complex consisting of AXIN, DVL, and GSK3B (Figure 1). This prevents the phosphorylation of β-catenin by GSK3B, and leads to its binding to the nuclear transcription factors, TCF7, LEF1, TCF7L1 and TCF7L2, leading to the activation of more than 60 different genes involved in growth regulation and differentiation, as well as GLP-1 expression [12]. Since Wnt signaling has a role in regulating and stabilizing β-catenin and its binding with TCF7L2, we hypothesized that any alternation in the canonical Wnt pathway would have profound consequences in insulin secretion and the generation of new β-cells, particularly given that Wnt signaling is required for normal development of the pancreas and islets during embryonic growth [13].

The present investigation is a follow-up study to explore the role of a novel, four-nucleotide (CCCA) insertion polymorphism we discovered in the most conserved region of *WNT16a* in US American Sikhs. The objectives of this investigation are: 1) to study the potential role of this *WNT16a* insertion in T2D in our diabetic sample of Punjabi Sikhs, 2) to quantify and compare gene expression of *WNT16a* and *TCF7L2* between carriers and non-carriers of the CCCA insertion within the *WNT16a* gene using mRNA samples from 27 frozen human pancreatic tissues, 3) to investigate the functional impact of this insertion on protein levels and message translation using a luciferase reporter vector containing the wild-type and mutant WNT16a 5′untranslated regions (UTR) transfected into cultured cells, and 4) to perform immunohistochemistry to examine the expression of WNT16a in human pancreas among insertion carriers vs. non-carriers.

## Methods

### Study participants

The DNA samples of 2,034 (52% male) individuals from our ongoing Sikh Diabetes Study (SDS) were used [14]. Of these, ~48% were ascribed as having T2D based on established guidelines of the American Diabetes Association, as described [15]. A medical record indicating either (1) a fasting blood glucose (FBG) *≥*126 mg/dL (*≥*7.0 mmol/L) after a minimum 12 h fast or (2) a 2 h post-glucose level (2 h oral glucose tolerance test [OGTT]) *≥* 200 mg/dL (*≥*11.1 mmol/L) on more than one occasion, combined with symptoms of diabetes, confirmed the diagnosis. Impaired fasting glucose (IFG) is defined as a fasting blood glucose level *≥*100 mg/dL (5.6 mmol/L) but *≤*126 mg/dL (7.0 mmol/L), as described previously [16]. Common characteristics observed in diabetics include excessive thirst, hunger, polyuria, blurry vision, common skin and urinary tract infections, nocturia, loss of bladder control, and fatigue. Impaired glucose tolerance (IGT) is defined as a 2 h OGTT >140 mg/dL (7.8 mmol/L) but <200 mg/dL (11.1 mmol/L). Subjects with IFG or IGT were considered pre-diabetics and were excluded from the analysis. The 2 h OGTTs were performed following the criteria of the World Health Organizations (WHO) (75 g oral load of glucose). Body mass index (BMI) was calculated as (weight (kg)/height (meter) [2]. Homeostasis Model Assessment (HOMA) for insulin resistance (HOMA-IR) was calculated as fasting glucose X fasting insulin/22.5, as described [17].

The normoglycemic subjects were recruited from the same Punjabi Sikh community and geographic location as the T2D patients [14]. The majority of the subjects were recruited from the state of Punjab in North India and Punjabi Sikhs living in the US. Individuals of South, East, and Central Indian origin were excluded, as were individuals with type-1 diabetes, a family member with type 1 diabetes, rare forms of T2D called maturity-onset diabetes of young (MODYs), or secondary diabetes (e.g., hemochromatosis, pancreatitis). Demographic and clinical characteristics of the SDS subjects are summarized in Table 1. All blood samples were obtained at the baseline visit and all participants provided a written informed consent for these investigations. All SDS protocols and consent documents were reviewed and approved by the University of Oklahoma Institutional Review Board and the Human Subject Protection Committees at the participating hospitals and institutes in India.

### Metabolic estimations

Insulin was measured by radio-immuno assay (Diagnostic Products, Cypress, USA). Serum lipids (total cholesterol, low density lipoprotein cholesterol [LDL-C], high-density lipoprotein [HDL-C], very low-density lipoprotein cholesterol [VLDL-C], and triglycerides [TG]) were measured by using standard enzymatic methods (Roche, Basel, Switzerland), as described [16,18]. C-peptide, TNFα, and MCP-1 measures were simultaneously quantified using Millipore’s Magnetic MILLIPLEX Human Metabolic panel (St. Charles, MO) and analyzed on a Bio-plex 200 multiplex system (Bio-Rad Hercules, CA), as described previously [19].

### Whole-genome exome sequencing

We performed genome-wide exome sequencing on two Punjabi Sikh subjects: a 64-year-old healthy normoglycemic male, and a 67-year-old diabetic female, using an Illumina GAIIx and “SureSelect Human All Exon Kit” by Agilent Technologies and “Paired-End Sequencing Library Prep by Illumina” (Version 1.0.1). The sequences containing 75x reads were filtered against public databases of genetic variants. The present investigation is focused on exploring the role of a frame-shift insertion (CCCA) discovered in a conserved region of human *WNT16a* gene (Additional file 1: Figure S1).

### Genotyping

Genotyping of the insertion polymorphisms was performed by polymerase chain reaction (PCR) and a gel-based assay. Forward primer Wnt16a-F (5') [TACCACTCTCCTCCCTCC] and reverse primer Wnt16a-R (3') [CCCTGATCAAATCCCCAAAT] were used to amplify the region containing the identified insertion; PCR amplification generated a 458 bp product in the sample containing no insertion. PCR conditions included an initial denaturation for 5 min. at 95°C, followed by 36 cycles (30 sec. 95°C, 45 sec. 53.7°C, 30 sec. 72°C), and a 10 min. extension at 72°C. Positive and negative controls were included for every PCR. 15μL of the PCR product was then separated on a 2.5% nusieve/agarose gel (3:1) for 2.5 hours at 140 volts to determine the genotype of participants as insertion (462 bp), non-insertion carriers 458 bp, and heterozygotes containing insertion/normal sequence of 462/458 bp (Additional file 1: Figure S2). To confirm the presence of the *WNT16a* insertion scored on the gel-based assay, approximately 30 samples were sequenced using an ABI 3730 capillary sequencer (Applied Biosystems Inc. Foster City, CA) and were analyzed using Mutation Surveyor DNA variant analysis software (v4.0.6.)(SoftGenetics, State College, PA)**.** Genotyping of rs7903146, located in intron 3 of the *TCF7L2* gene, was performed with a TaqMan genotyping assay (Applied Biosystems, Foster City, CA), using a 7900 genetic analyzer, as described previously [4].

### Quantitative gene expression studies on WNT16a

Gene expression studies for Wnt16a were performed using 27 human pancreatic tissue specimens (13 diabetic and 14 non-diabetics) collected from the Department of Surgery at the University of Oklahoma Health Sciences Center. Total RNA was extracted from frozen tissues (stored in liquid nitrogen) using Ambion’s mirVana RNA kits (Grand Island, NY), followed by RT-PCR using Bio-Rad’s iScript RT-PCR kit (Hercules, CA), according to the manufacturers’ instructions. Real Time PCR was then performed using an ABI 7900HT genetic analyzer in conjunction with Qiagen’s QuantiTect primer assay (Chatworth, CA) and Bio-Rad’s iTaq SYBR Green Supermix with ROX (Hercules, CA). Results were then analyzed on ABI’s RQ Manager (v.1.2.1) software. Beta-actin was used as a normalizing control.

### Transient DNA transfection and dual-luciferase assay

The 5′ UTRs of the wild-type and mutant Wnt16a message were incorporated into oligonucleotide primers as depicted in Additional file 1: Figure S3. Note that each of the 5′ primers incorporated a Sac I site for insertion into pCI-GFP, followed by the sequence of the Wnt16a 5′ UTR, then a region homologous to firefly luciferase. The pCI-GFP vector was developed by inserting eGFP into the parent vector, pCI-Neo (Promega, Madison, WI), and allowed us to monitor transfection efficiency. The 3′ primer was homologous to a site in the pGL3 vector past a unique Xba I site in the vector. After PCR amplification using pGL3 as a template, the amplimers were digested with Sac I and Xba I, and then ligated into pCI-GFP. For transfection into cultured cells, each construct (0.125 μg per culture well) was added to 1 μl Plus reagent and 15 μl Opti-MEM (Life Technologies, Carlsbad, CA), along with 0.125 μg per well of an empty pGL3-Basic vector (which served as carrier DNA) and 0.01 μg per well pGL4.74 (a Renilla luciferase construct used for normalization) for a total of 0.26 μg DNA. This was added to 0.5 μl Lipofectamine reagent in an additional 15 μl of Opti-MEM and used to transfect HEK-293 cells (74,000 cells per well) in a 48-well plate. After 48 hours in medium plus 10% calf serum, cells were washed in PBS, and lysed for luciferase activity. Lysates were diluted until the luciferase values fell within a linear response range. Both firefly and *Renilla* luciferase values were measured using a dual luciferase detection kit (Promega, Madison, WI).

### Immunohistochemistry

Formalin-fixed paraffin-embedded pancreatic tissues were cut at a thickness of 4 μm, mounted on SuperfrostPlus® slides (Statlab Medical Products, Lewisville, TX), and subsequently deparaffinized, rehydrated, and washed in Tris Buffered Immunohistochemistry wash buffer + Tween 20 (TBST, catalog# 935B, Cell Marque, Rocklin, CA). Antigen retrieval was accomplished by placing slides in 10 mM citrate buffer, pH 6.0 (cat. #S2389,Target Retrieval Solution, DAKO, Carpentaria, CA), in a steamer for 20 minutes, followed by 20 minutes cooling in deionized water at room temperature. According to the manufacturer’s directions, sections were treated with a background blocker (cat. #927B, Cell Marque, Rocklin, CA) and a peroxidase blocking reagent (cat. #925B, Cell Marque, Rocklin, CA) to inhibit endogenous peroxidase activity, followed by three, five-minute washes each in deionized water. Rabbit anti-Wnt antibody was prepared in antibody diluent (cat. #936B, Cell Marque, Rocklin, CA) and added to slides at 2 μg/ml (1:500 dilution, cat. #LS-A9630, MBL International Corporation, Woburn, MA). Antigen retrieval was accomplished according to the manufacturer’s recommendation for the Wnt16 antibody (LSBio, Woburn, MA). Following incubation for 1 hr at room temperature, the sections were processed for immunohistochemistry using the HiDef detection HRP Mouse/Rabbit polymer system (cat. #954D, Cell Marque, Rocklin, CA). Sections were washed three times for five minutes each in tris buffered immunohistochemistry wash buffer + Tween 20 (TBST), incubated with the amplifier, washed three times for five minutes each in TBST, and incubate with labeled polymer. Following a final wash in TBST, slides were incubated with 3′3′diaminobenzidine tetrahydrochloride (DAB) (cat. #957D, DAB substrate Kit, Cell Marque, Rocklin, CA). Counterstaining was performed with Immuno^*^ Master Hematoxylin (American Master*Tech Scientific, Inc., Lodi, CA). Controls were incubated with rabbit IgG isotype at 2 μg/ml (rabbit [DA1E] mAB IgGXP® isotype control, cat. #3900, Cell Signaling Technologies Danvers, MA). A total of seven tissues (1 T2D and 6 controls) with *Wnt16a* genotypes were used for immunohistochemistry. Slides were scored based on intensity (0- no, 1- weak, 2- moderate and 3- strong), and the area of stain (0 for 0%, 1-<10%, 2-between 10-15%, and 3- between 51-81%). The consolidated scores (ranging from 0–7) were derived from the sum of scores of intensity and area, negative being in the range of 0–2, weakly positive-3, moderately positive ranging from 4–5, and highly positive ranging from 6–7.

**Table 1:** **Clinical characteristics of study subjects stratified by *Wnt16a *** **insertion carriers versus non-carriers**

**Trait**	**Non carrier**	**Carrier**	***P *** **value**
Number	1377	657	-
% Males	52	51	-
Age (yrs)	53.4 ± 12.9	51.7 ± 12.1	0.004
**Obesity**			
BMI (kg/m^2^)	26.8 ± 4.9	26.8 ± 5.1	0.930
Weight (kg)	69.8 ± 14.0	70.0 ± 14.3	0.811
Waist (cm)	93.6 ± 12.2	93.4 ± 12.0	0.704
WHR	0.95 ± 0.08	0.95 ± 0.08	0.242
**Metabolic**			
Fasting Blood Glucose mg/dL	120.7 ± 45.4	121.5 ± 45.4	0.736
Insulin (μIU/mL)	6.9 (6.6-7.3)	7.5 (7.0-8.1)	0.081
HOMA-IR	2.0 (1.9-2.2)	2.2 (2.1-2.4)	0.059
C-peptide pg/mL	519.5 (473.7-569.7)	602.8 (525.6-691.4)	0.078
**Inflammation**			
TNFα pg/mL	7.9 (7.4-8.5)	9.2 (8.2-10.2)	0.029
MCP1 pg/mL	315.5 (296.7-335.6)	326.5 (297.9-357.8)	0.541
**Lipid**			
Triglyceride mg/dL	149.0 ± 82.3	151.5 ± 85.9	0.542
Total Cholesterol mg/dL	173.8 ± 52.9	179.2 ± 49.9	0.038
HDL-C mg/dL	37.2 ± 14.7	37.9 ± 14.2	0.292
LDL-C mg/dL	102.1 ± 40.0	104.9 ± 38.3	0.143

WHR-Waist to hip ratio, LDL-C -low density lipoprotein cholesterol, HDL-C -high density lipoprotein cholesterol, BMI- body mass index, HOMA-IR -homeostasis model assessment for insulin resistance.

### Statistical analysis

#### Association analysis

Data quality for SNP genotyping was checked by establishing reproducibility of control samples. Departure from Hardy-Weinberg equilibrium in controls was checked using Pearson’s Chi-square, as reported previously [5]. Descriptive statistical analyses were performed with SPSS Statistics Software (v 15.0). The chi-square test for categorical variables and t-test for continuous variables were used to test differences where appropriate. While multivariate logistic-regression was used to assess the association of the insertion with T2D and obesity, multivariate linear-regression was used for each quantitative trait after adjustment for relevant covariates (age, sex, diabetes status, BMI, and medication), assuming an additive model. Skewed variables were detected by Shapiro-Wilk’s test for continuous traits. Subsequently, TG, total cholesterol, LDL-C, VLDL-C, FBG, C-peptide, MCP-1, and HOMA-IR were normalized by log-transformation before statistical comparisons, and all p-values were derived from analyses of transformed data. The summary statistics (β, S.E., and p-values) were used to assess SNP-phenotype association. Gene expression analyses were performed using Applied Biosystems’ RQ Manager (v.1.2), which uses the comparative C_T_ method for relative quantification. We determined the ΔC_T_ value by (Target Average C_T_-Endogenous Control Average C_T_), then calculated the ΔΔC_T_ to determine the fold-difference in gene expression by ΔC_T_ Target - ΔC_T_ Calibrator. For the amount of target determination, the data were normalized to the endogenous control and relative to the calibrator by using 2^-ΔΔCT^ as described [20] . For reporter assays, the results are presented as the mean ± average deviation from the mean for the number of observation, as indicated. Statistical significance of differences between groups was estimated using a two-tailed *t* test.

## Results

### Whole-exome sequencing

As summarized in Additional file 2: Table S1, a total of 20,306 mutations were found in the control and 21,258 in the diabetic subjects. Among these, 4,673 and 4,842 novel SNPs were uniquely present in control and T2D cases, respectively. To identify the functional significance of the variants identified, we performed initial comparative genomic screening on the mutations found in some selected loci using UCSC’s Vista Genome Browser. From these results, several candidate genes involved in insulin secretion, β-cell proliferation, or related pathways were identified (data not shown). Interestingly, novel substitution in *WNT16a,* which showed a 4-base-pair frame-shift insertion near two known SNPs, was in an evolutionarily conserved region (as shown in Additional file 1: Figure S3) and was predicted to be disruptive.

### Association studies

**Figure 2:**
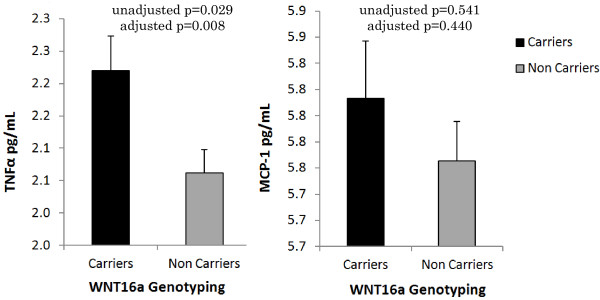
**Distributions of serum levels (mean ±SD) of inflammatory cytokines (TNFα and MCP-I) among Wnt16a insertion carriers non-carriers in SDS subjects.** Serum levels of TNFα were significantly higher (p=0.008) in insertion carriers, while a similar but non-significant trend was seen with MCP-1. The statistical analysis was performed in combined samples (T2D and controls) after adjusting for the confounding effects of age, BMI, gender, and T2D status.

A genetic screening of 2,034 SDS individuals (977 T2D cases and 1,057 controls T2D cases and 1,057 controls) showed that 33% of T2D cases and 32% non-diabetic controls were carriers of a CCCA insertion; the number of carriers of this insertion did not differ significantly among cases versus controls (p=0.08). Multiple regression analysis, performed in diabetics and non-diabetic controls separately, did not reveal any association of the *Wnt16a* insertion with obesity (BMI, waist-to-hip ratio [WHR]) (Table 1). However, the insertion carriers showed moderately higher mean (±SD) levels of total cholesterol compared to non-carriers (173.8±52.9 (mg/dL) vs. 179.2±49.9 (mg/dL), p=0.038). Serum mean levels of inflammatory cytokines TNFα were also significantly higher among insertion carriers compared to non-carriers (p=0.008) (Figure 2). A similar but non-significant trend was seen with increased mean levels of MCP-1 among insertion carriers compared to non-carriers (p=0.440) (Figure 2). There was a significant difference in the frequency of ‘T’ (the at risk allele for T2D) in rs7903146 of *TCF7L2* among cases and controls (38% cases vs. 28% controls). The age- and sex-adjusted OR showing ‘T’ allele-associated T2D risk was 1.51 (95%CI [1.37-1.66], p=1.53x10^-17^). However, no association of *TCF7L2* polymorphism was seen with inflammatory cytokines (TNFα or MCP-1) (data not shown).

### Bioinformatics, gene expression studies, and western blotting

**Figure 3:**
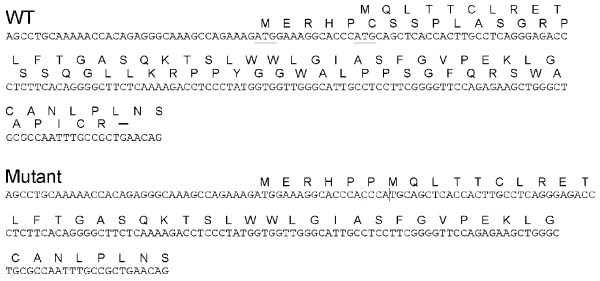
**The human *Wnt16 *gene includes two alternative transcription start sites, resulting in two alternative first exons and three common exons.** The Wnt16a message, which is only expressed in pancreas, includes an upstream open reading frame (uORF) that initiates 14 bp 5′ of the coding sequence AUG. As shown, in the *Wnt16a* wild-type allele, translation of the 5′ UTR would terminate 140 base pairs later, presumably resulting in nonsense-mediated decay, since two down-stream exon junction complexes would not be disrupted during the pioneer round of translation. The 4 base-pair CCCA insertion of the mutated Wnt16a message, on the other hand, results in the transition of the uORF to an in-frame alternative translation initiation site. Translation initiation from either AUG during the pioneer round of translation would not trigger nonsense-mediated decay of the Wnt16a massage.

**Figure 4:**
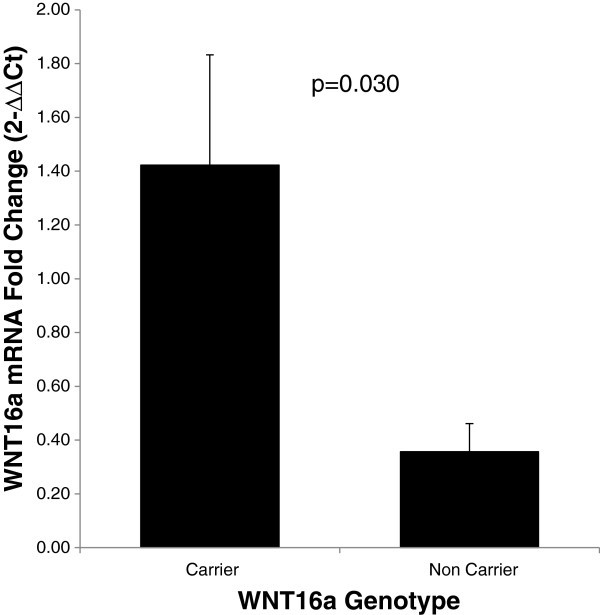
**Gene expression studies for Wnt16a were carried out using 27 pancreas tissue samples by quantifying mRNA expression of WNT16a by real-time PCR.** Of 27 participant donors of human pancreatic tissue, nine were carriers of CCCA insertion in *WNT16a*. Our data revealed a 3.2 fold increase in the expression of WNT16a among insertion carriers compared to non-carriers.

**Figure 5:**
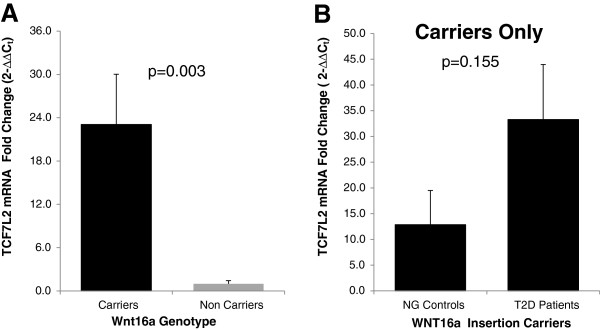
**Gene expression study of TCF7L2 in the same 27 pancreatic tissues used to determine the expression of Wnt16a by real-time PCR analysis.** Figure 5A shows a significant elevation of TCF7L2 mRNA levels among CCCA insertion carriers and a very low expression of TCF7L2 mRNA was observed in non-carriers (p= 0.003). Figure 5B shows that within CCCA insertion carriers, the expression of TCF7L2 mRNA in pancreas was elevated among diabetics compared to non-diabetic controls.

**Figure 6:**
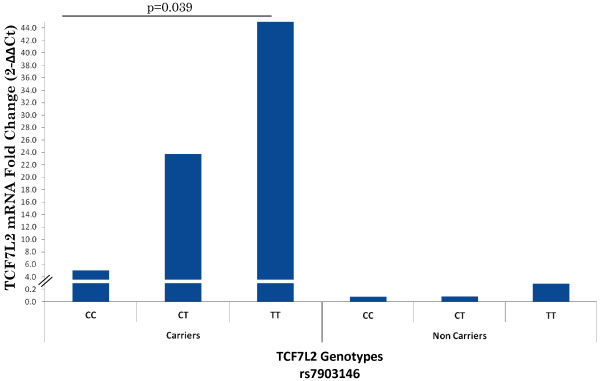
**Stratification of TCF7L2 mRNA quantitation by *TCF7L2 *genotypes of rs7903146 among Wnt16a insertion carriers and non-carriers.** The at-risk ‘T’ allele carriers of *TCF7L2* with CT+TT genotypes showed a 8.7-fold increase in the expression of TCF7L2 compared to CC genotypes. Note that this increase was only observed in *Wnt16a* (CCCA) insertion carriers and not in the non-carriers.

The Wnt16a message, which is uniquely expressed in pancreas, includes an upstream open reading frame (uORF) that initiates 14 bp 5′ of the coding sequence AUG (Figure 3). Translation of this 5′ UTR would terminate 140 bp later, presumably resulting in nonsense-mediated decay (NMD) of the message, since two down-stream exon junction complexes would not be disrupted during the pioneer round of translation [21]. The 4-base-pair insertion (CCCA) of the mutated Wnt16a message, on the other hand, would result in the transition of the uORF to an in-frame alternative translation initiation site. In this case, translation initiation from either the first or second AUG during the pioneer round of translation would not trigger NMD. RT-PCR and qualitative gene expression studies were performed by quantifying mRNA expression of *WNT16a* and *TCF7L2* genes among carriers and non-carriers of Wnt16a. Of 27 participant donors of human pancreatic tissue, nine were carriers of the insertion in *WNT16a*. As shown in Figure 4, our data revealed a ~3.2-fold increase in the expression of WNT16a among insertion carriers compared to non-carriers. The expression of WNT16a was consistently higher among insertion carriers irrespective of disease status. Gene expression analysis of *TCF7L2* in the same pancreatic tissues revealed a significant elevation (p=0.003) of the amount of TCF7L2 mRNA among insertion carriers compared to non-carriers (Figure 5A). In the stratified data by disease within CCCA insertion carriers, the expression of TCF7L2 mRNA was higher in diabetic pancreatic tissues compared to non-diabetic pancreas, however, this increase was not statistically significant (p=0.155). (Figure 5B). Further stratification of quantitative mRNA expression among the at-risk ‘T**’** allele carriers of *TCF7L2* SNP (rs7903146) revealed that the CT+TT genotypes showed an 8.7-fold increase in the expression of TCF7L2 compared to CC genotypes in Wnt16 a insertion carriers, while the non-insertion carriers showed the same allelic trend at a significantly reduced magnitude (Figure 6).

### Luciferase reporter assay

**Figure 7:**
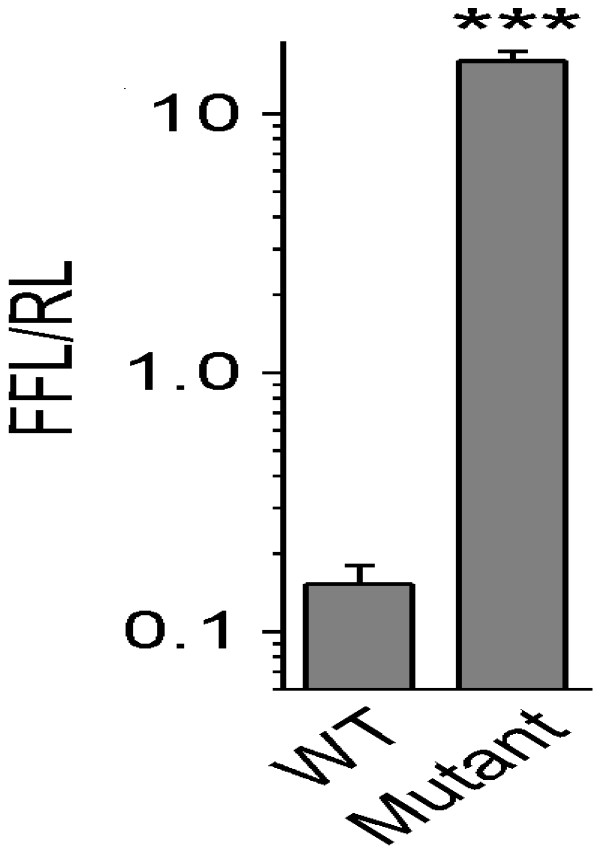
**Reporter constructs assembled with the mutated form of the Wnt16a 5′ UTR are more efficiently translated than the wild-type form.** The wild-type and mutant Wnt16a 5′ UTR sequences were inserted adjacent to a luciferase cDNA, and the resulting plasmids were used to transfect HEK-293 cells. 48 hours after transfection, cells were lysed, and firefly luciferase (FFL) and *Renilla* luciferase (RL) levels were measured. Our reporter constructs using the wild-type and the mutant (insertion) sequence of Wnt16a showed significantly increased (70%, p<0.0001) levels of luciferase protein in the constructs carrying the mutant sequence.

In order to further evaluate the influence of the CCCA insertion on translation of the Wnt16a message, we assembled reporter constructs driven by the cytomegalovirus (CMV) promoter that included the wild-type and the mutant sequence of the Wnt16a 5′ UTRs. The long uORF was mimicked in our luciferase construct by the presence of a translation stop site in-frame with the upstream AUG. If the first AUG in the message was used to initiate translation, then no luciferase protein should have been produced. Indeed, when we inserted the additional four nucleotides to replicate what occurs in the mutant situation, we noted a significantly increased level (~70%) of luciferase expression (p=0.0001) (Figure 7). This suggests that the upstream AUG can act as an efficient translation initiation site. In the wild-type gene, this would reduce expression of the full-length protein by preventing initiation at the second AUG. In the presence of the CCCA insertion, both AUGs are in the same reading frame, so full-length protein would be produced regardless of which AUG was used to initiate translation.

### Immunohistochemistry

**Figure 8:**
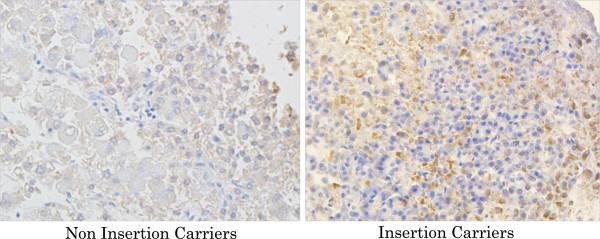
**Immunoperoxidase staining of 5 micron thick histological sections of paraffin-embedded pancreatic tissues.** The sections were processed for immunohistochemistry using the HiDef detection HRP Mouse/Rabbit polymer system as described in detail under methods. The CCCA insertion carriers revealed a higher expression of Wnt16, showing staining score from +1 to +3 among insertion carriers versus negative staining in non-carriers, which showed staining scores from 0 to +1. The scoring intensity was not different among diabetic and non-diabetics.

Immunoperoxidase staining of paraffin-embedded pancreatic tissues of normoglycemic controls and diabetic cases were scored for the intensity of antibody as described in methods. As shown in Figure 8, the tissues with insertion carriers revealed a higher expression of Wnt16a showing high intensity staining among insertion carriers verses negative staining in non-carriers. The scoring intensity was indifferent among diabetics and non-diabetics.

## Discussion

The key effector pathway of Wnt signaling (*β*-cat/TCF7L2) has been recently implicated in metabolic homoeostasis, diabetes, obesity, osteoporosis, cardiovascular disease, and cancer [9,22-24]. The discovery of *TCF7L2* as a T2D susceptibility gene in different ethnic populations through genome-wide studies has triggered numerous investigations to explore the clinical utility of identifying *TCF7L2* genetic variations, and whether the identified SNPs can be used as markers for tailoring customized therapeutics. However, the underlying molecular mechanism by which *TCF7L2* variants influence T2D remains unclear. While a number of recent studies have suggested the essential involvement of *β*-cat/TCF7L2 in the Wnt signaling pathway for pancreatic development and function [25,26], the role of *β*-cat in pancreatic *β* cell development remains unclear and controversial [13,27]. Mice lacking *β*-cat developed pancreatitis prenatally; however, they later recovered from pancreatitis and regenerated normal pancreas and duodenal villi from wild-type cells that escaped earlier *β*-cat deletion. These observations suggested that mouse embryos were capable of overcoming substantial *β*-cat reduction through complicated compensatory mechanisms [13]. Other studies have shown that the over-expression of *β*-cat at different development stages generated different effects [27]. Similarly, some studies suggest an essential and beneficial role of TCF7L2 in pancreatic β cell development [28,29], while other studies revealed a destructive role of TCF7L2 by over-expression of TCF7L2 mRNA due to alternatively spliced variants, which increased the risk of developing T2D [30]. Further, the increased expression of TCF7L2 in pancreatic β-cells was positively correlated with insulin gene expression but was negatively correlated with glucose-stimulated insulin release [30]. Therefore, it is still unclear how *β*-cat/TCF in Wnt signaling is mechanistically involved in pancreatic development and increased T2D susceptibility.

In this investigation, the discovery of a frame-shift insertion in the most conserved region of *WNT16a* (Additional file 1: Figure S4), and the restricted and exclusive expression of Wnt16a isoform in the human pancreas [31], prompted us to explore the role of this *Wnt16a* insertion in T2D using genetic epidemiologic, molecular, and physiologic studies. *TCF7L2* polymorphisms have demonstrated the biggest effect on the risk for developing T2D in recent GWAS and replication studies in multiple ethnic populations, including our own studies in Asian Indians [4-6,32,33]**.** The Wnt16a isoform is exclusively expressed in the pancreas of humans, while its close relative, Wnt16b, is ubiquitously expressed in many other organs [31]. The prevalence of the CCCA insertion polymorphism did not differ significantly among diabetic cases (33%) versus controls (32%) in our cohort. Although our epidemiological data did not clarify the role of CCCA insertion in T2D, obesity, or lipid metabolism (Table 1), our multiple linear regression results showed significant elevation in serum TNFα levels among insertion carriers versus non-carriers (p= 0.008), as well as a non-significant trend in the same direction for another inflammatory marker, MCP-1 (p=0.44). These findings are in agreement with earlier studies reporting the influence of Wnt signaling in inflammation [34], and suggest that the presence of the CCCA insertion appears to promote circulatory levels of pro-inflammatory cytokines in our samples.

Our *in silico* analysis (Figure 3) clearly suggested that the frame-shift insertion of the mutated WNT16a results in the transition of the uORF to an in-frame alternative translation initiation site. During the pioneer round of translation, initiation at this up-stream AUG would not result in NMD. In non-carriers, initiation at this up-stream AUG would prevent the production of mature protein, and would likely result in NMD, thereby reducing the expression of this gene. This was further verified in our quantitative real-time PCR results that consistently showed the wild-type (non-insertion carriers) message levels being ~3.2-fold lower than those observed in samples from the insertion carriers (Figure 4). Additional evidence of the influence of the CCCA insertion on translation of the message was obtained using reporter constructs that incorporated the wild-type and the mutant (insertion) sequence of the WNT16a 5′ UTR. Using this approach, we noted a marked increase in the levels of luciferase expression in the constructs carrying insertion (p=0.0001) (Figure 7). This was additionally confirmed in histological sections of the embedded human pancreatic islets stained with Wnt16 antibody. It was interesting to observe that the tissues with insertion carriers showed higher expression of Wnt16a with staining score ranging from +1 to +3 verses negative staining in non-carriers (Figure 8).

Our comparison of the expression of TCF7L2 mRNA in the same pancreatic tissues used for Wnt16a analysis showed a significantly increased (p=0.003) expression of TCF7L2 among the *WNT16a* insertion carriers compared to the wild-type (non-carriers) (Figure 5A). This significantly enhanced expression of Wnt16a and TCF7L2 among insertion carriers in human pancreas would be predicted to affect the expression of several *β*-cat /TCF7L2 or Wnt downstream target genes [22]. It was interesting to observe that, despite the fact that the frequency of the at-risk ‘T’ allele in rs7903146 of *TCF7L2* did not differ among *WNT16a* insertion and non-carriers (0.34 insertion carriers vs. 0.33 non-carriers), TCF7L2 mRNA levels were significantly elevated (~23 folds) among *WNT16a* insertion carriers vs. non-carriers (Figure 5A). Additionally, the at-risk ‘T’ allele carriers of *TCF7L2* (rs7903146) also showed significantly increased expression of TCF7L2 mRNA in pancreas compared to CT and CC carriers (Figure 6). This is consistent with enhanced Wnt signaling, something we would predict given the impact of the *Wnt16a* insertion mutation identified here.

TCF7L2 has been shown to be abundantly expressed in GLP-1-producing intestinal epithelial cells [35]. It has also been shown to be expressed in pancreas and to mediate pancreatic β cell proliferation and survival [28,36]. However, in other studies, TCF7L2 was shown to be present at low levels or not expressed at all in pancreas [29,35,37]. We have identified a significant elevation of TCF7L2 mRNA in pancreas, especially among the CCCA insertion carriers, which appears to increase diabetes risk by increasing the expression of TCF7L2 among ‘T’ risk allele carriers of rs7903146 of *TCF7L2.* These results suggest a synergistic effect of *Wnt16a* insertion and the at-risk ‘T ’allele of *TCF7L2* in compounding the risk of T2D, likely through elevated β-cat/TCF7L2 activity and the expression of downstream Wnt targets. Higher expression of TCF7L2 among ‘T’ allele carriers was evident in pancreatic tissues of diabetic patients compared to non-diabetic controls. These results are in agreement with earlier findings by Lysenko et al. [30], where carriers of ‘T’ allele in rs7903146 of *TCF7L2* exhibited five-fold increases in TCF7L2 mRNA levels in pancreatic islets of diabetic patients, and showed an associated impairment of insulin secretion. Previous findings by others have shown that, while elevated mRNA expression of TCF7L2 was linked with ‘T’ risk allele of rs7903146, even though no apparent increase in TCF7L2 protein amount was observed [38,39]. In spite of this, the same groups demonstrated that the higher mRNA expression of TCF7L2 variants resulted in the down-regulation of GLP-1-induced insulin secretion, and increased the risk of T2D through Wnt signaling [38,40]. Since GLP-1 receptors are primarily located in pancreas and Wnt16a is exclusively expressed in pancreas, it is quite conceivable common insertion polymorphism in *WNT16a* may affect GLP-1 receptor activity by modulating TCF7L2 expression, thus influence GLP-1-induced insulin secretion. Since Wnt signaling is known to stabilize the binding of β-catenin with TCF7L2, which is critical for expression of many other genes involved in β-cell development, any alteration in the canonical Wnt pathway should have profound consequences in insulin secretion and the generation of new β-cells, as this pathway is required to be tightly regulated. It will be also of interest to determine if WNT16a can modulate GLP-1 receptor expression independent of TCF7L2.

## Conclusions

To our knowledge, ours is the first study reporting the role of WNT16a in *β*-cat/TCF7L2 signaling and the risk of developing T2D, which appears to be mediated through the increased expression of TCF7L2 in pancreas, a pathway critical for the regulation of several dozen downstream genes involved in glucose metabolism, apoptosis, skeletal muscle function, and atherosclerosis. Therefore, a detailed examination of Wnt16a and its potential role in genetic predisposition to T2D through Wnt signaling, and cross-talk between other signaling pathways, may help identify therapeutic targets for the treatment of T2D.

## Competing interests

We declare that there is no conflict of interests that could be perceived as prejudicing the impartiality of the research reported.

## Author contributions

Conceived and designed the experiments: DKS; Provided pancreatic tissues and immunohistochemistry: DB, ML, SL; Western blotting and luciferase studies: EWH, ECB; Genotyping, gene expression and analysis: LFB; Contributed reagents/materials/analysis tools: DKS and EWH; Wrote the paper: DKS and EWH; Guarantors: DKS, EWH. All authors read and approved the final manuscript.

## Supplementary Material

Additional file 1: Figure S1 Exome sequencing reveals the presence of 4 base pair insertion (CCCA) between A and T of ATG start codon in Wnt16a which was confirmed by targeted sequencing of 30 DNA samples using an ABI 3730 sequencer (Applied Biosystemes Inc. Foster City, USA) and were analyzed using Mutation Surveyor (v4.0.6.). **Figure S2.** Nusieve-Agarose (3:1) gel showing wild-type (458 bp), heterozygous insertion (458/462 bp) and homozygous insertion (462 bp) bands in *WNt16a* gene. **Figure S3.** Oligonucleotides used to amplify Wnt-16a luciferase reporter constructs. **Figure S4.** Comparative genomic analysis showing evolution of translation initiation sites in Wnt16a. Arrow indicates the position of insertion in the evolutionarily conserved region at the start codon. Click here for file

Additional file 2: Table S1 Genome-wide Exome Sequencing in Asian Sikhs. Click here for file
