# Supplementary material for: Carriers of a novel frame-shift insertion in WNT16a possess elevated pancreatic expression of TCF7L2
Source: BMC Genet. 2013 Apr 23;14:28. doi: 10.1186/1471-2156-14-28 (PMC3675375; doi:10.1186/1471-2156-14-28)
Supplement: Additional file 1: Figure S1 — Exome sequencing reveals the presence of 4 base pair insertion (CCCA) between A and T of ATG start codon in Wnt16a which was confirmed by targeted sequencing of 30 DNA samples using an ABI 3730 sequencer (Applied Biosystemes Inc. Foster City, USA) and were analyzed using Mutation Surveyor (v4.0.6.). Figure S2. Nusieve-Agarose (3:1) gel showing wild-type (458 bp), heterozygous insertion (458/462 bp) and homozygous insertion (462 bp) bands in WNt16a gene. Figure S3. Oligonucleotides used to amplify Wnt-16a luciferase reporter constructs. Figure S4. Comparative genomic analysis showing evolution of translation initiation sites in Wnt16a. Arrow indicates the position of insertion in the evolutionarily conserved region at the start codon. [file 1471-2156-14-28-S1.docx]

**Supplementary Data**

**Figure S1**


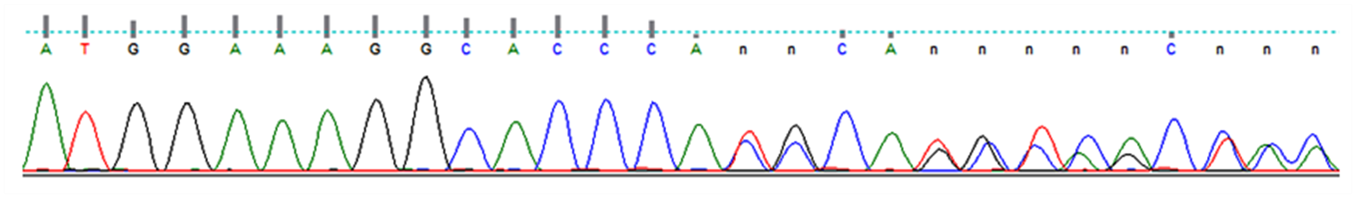


**C C C** **A**


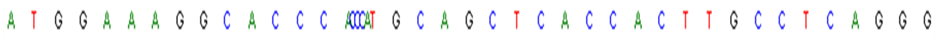

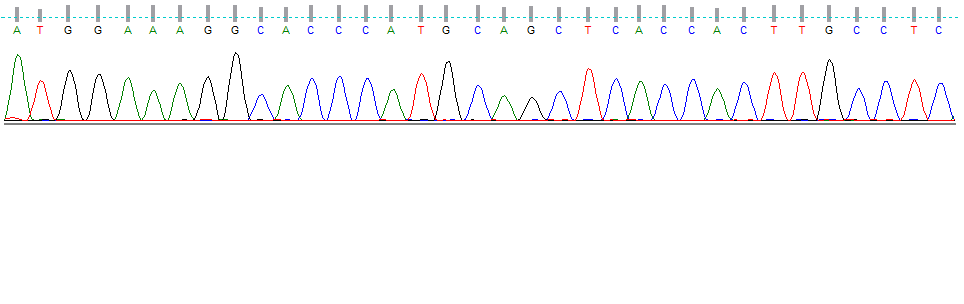


Wild-Type

Insertion

**Figure S2**

**
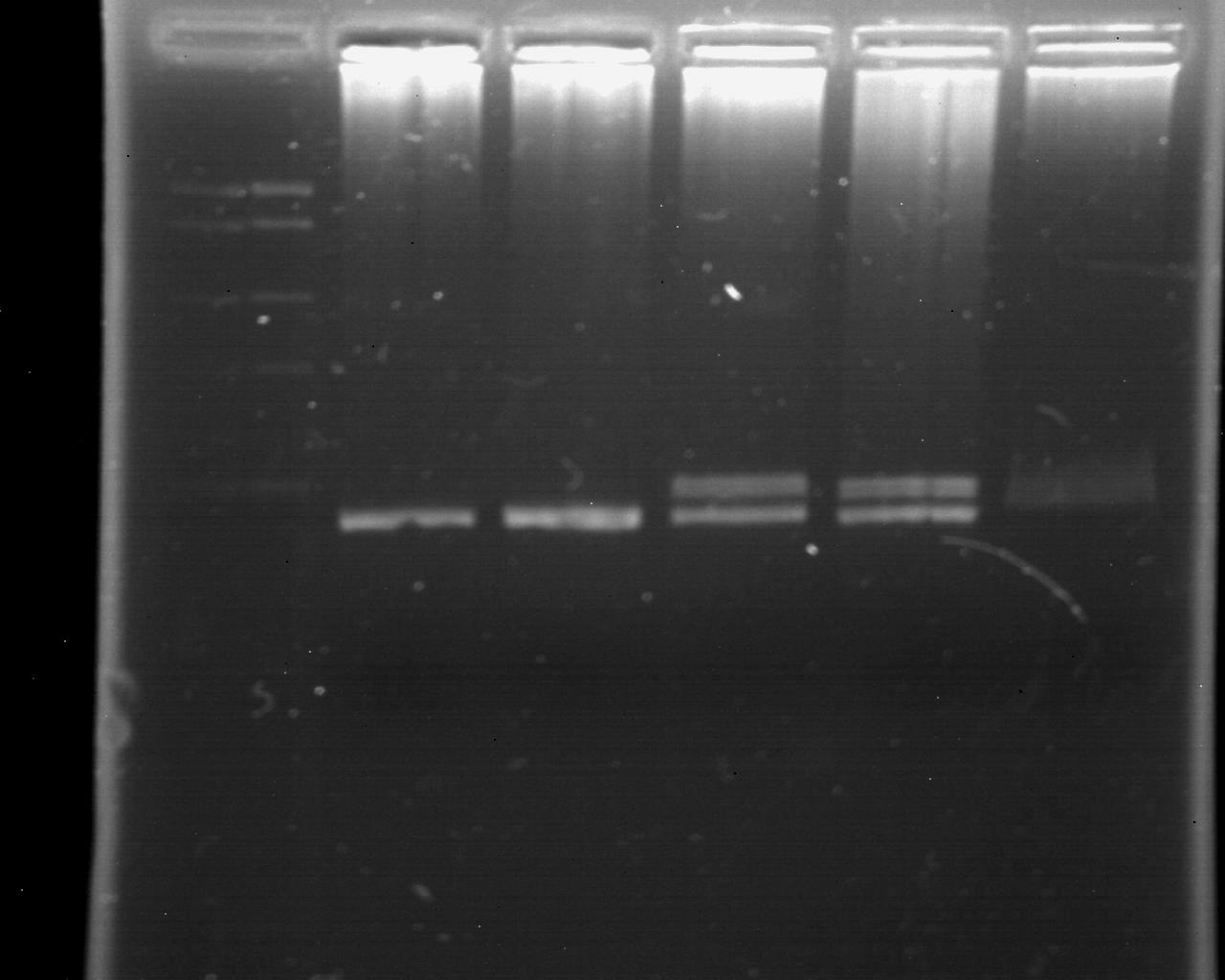
**

Non Carrier

Non Carrier

Heterozygous Carrier

Heterozygous Carrier

Homozygous Carrier

**Figure S3**


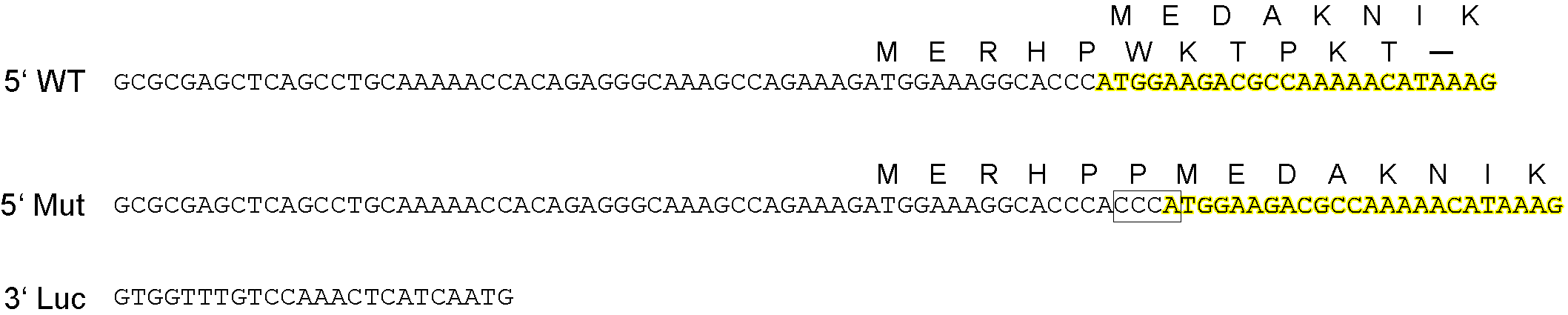


**Figure S4**

Human **ATG**GAAAG[G](http://uswest.ensembl.org/Homo_sapiens/ZMenu/TextSequence?db=core;factorytype=Location;g=ENSG00000002745;r=7:120965421-120981158;t=ENST00000361301;v=rs10668066;vdb=variation;vf=24950714)[C](http://uswest.ensembl.org/Homo_sapiens/ZMenu/TextSequence?db=core;factorytype=Location;g=ENSG00000002745;r=7:120965421-120981158;t=ENST00000361301;v=rs10668066;v=rs34393077;vdb=variation;vf=24950714;vf=25140174)[A](http://uswest.ensembl.org/Homo_sapiens/ZMenu/TextSequence?db=core;factorytype=Location;g=ENSG00000002745;r=7:120965421-120981158;t=ENST00000361301;v=rs34393077;vdb=variation;vf=25140174)CCC**ATG**CAGCT

Chimpanzee **ATG**GAAAGGCACCC**ATG**CAGCT

Gorilla **ATG**GAAAGGCACCC**ATG**CAGCT

Guinea Pig **AAT**GAAATGCATCC**ATG**CAGCC

Mouse **ATG**GAAGCCTGTCC**CAG**CAAAC

Rat **ATG**GAAGCCTGCCC**CAG**GAAAC

Panda Bear **AGG**GCAGGAAGTCC**ATG**CAGAC

Dog **---**-------GTCC**ATG**CAGAG

Bat **ATG**GAAGGGCATAC**ATG**-AGAC

Dolphin **ATG**GAAGGGCATCC**ACG**CAGAC

Alpaca **AAC**AGGGGGCCTCC**AGG**AAGCC

Boar **AGA**GAAGCCGGTCC**CTG**TGGAC

Hedgehog **---**-AAGGATATCC**ATG**CAGAA

**Legends (Supplementary Figures)**

**Figure S1.** Exome sequencing reveals the presence of 4 base pair insertion (CCCA) between A and T of ATG start codon in Wnt16a which was confirmed by targeted sequencing of 30 DNA samples using an ABI 3730 sequencer (Applied Biosystems Inc. Foster City, USA) and were analyzed using Mutation Surveyor (v4.0.6.).

**Figure S2.** Wnt 16a PCR fragments in lanes 1-2 containing wild type 458 bp ( homozygous), in lanes 3-4 (heterozygous carriers) 458/462 bp, and lane 5 (homozygous carrier) 462 bp.

**Figure S3.** Oligonucleotides used to amplify Wnt-16a luciferase reporter constructs.

**Figure S4.**  Comparative genomic analysis showing evolution of translation initiation sites in Wnt16a. Arrow indicates the position of insertion in the evolutionarily conserved region at the start codon.
