# Supplementary material for: Carriers of a novel frame-shift insertion in WNT16a possess elevated pancreatic expression of TCF7L2
Source: BMC Genet. 2013 Apr 23;14:28. doi: 10.1186/1471-2156-14-28 (PMC3675375; doi:10.1186/1471-2156-14-28)
Supplement: Additional file 2: Table S1 — Genome-wide Exome Sequencing in Asian Sikhs. [file 1471-2156-14-28-S2.doc]

| **Table 1S. Genome-wide Exome Sequencing in Asian Sikhs** | | | |
| --- | --- | --- | --- |
|  | Control | | Case |
| Individual Mutations | 20306 | | 21258 |
| Individual SNPs | 19614 | | 20583 |
| Individual Deletions | 401 | | 380 |
| Individual Insertions | 291 | | 295 |
| Novel Mutations | 4942 | | 5127 |
| Novel SNPs | 4673 | | 4842 |
| Novel Deletions | 156 | | 169 |
| Novel Insertions | 113 | | 116 |
| % of total mutations shared | | 54 | |
|  | | | |
